# The first complete mitochondrial genome of *Phellinus pomaceus* var. *prunastri* (Pers.) Pat. 1926 (Hymenochaetales: Hymenochaetaceae) and phylogenetic analysis

**DOI:** 10.1080/23802359.2024.2438275

**Published:** 2024-12-08

**Authors:** Wei Gao, Shuyi Chen, Qiang Li

**Affiliations:** a Clinical Medical College & Affiliated Hospital of Chengdu University, Chengdu University, Chengdu, Sichuan, China; b School of Food and Biological Engineering, Chengdu University, Chengdu, Sichuan, China

**Keywords:** Mitochondrial genome, fungi, evolution, phylogeny

## Abstract

*Phellinus pomaceus* var. *prunastri* (Pers.) Pat. 1926 is a famous medicinal fungus that has attracted considerable interest in biotechnology because of its diverse biologically active ingredients. Here, we provide the full mitochondrial genome sequence of *P. pomaceus*, which spans 122,850 bp and has a GC content of 26.04%. The genome comprises 15 essential protein-coding genes, 26 distinct ORFs, 24 intronic ORFs, 25 tRNAs, and 2 rRNA genes. Bayesian inference (BI) was employed for phylogenetic analysis, revealing the evolutionary relationships among 17 Basidiomycota fungi. The results strongly supported distinct clades and indicated that *P. pomaceus* is closely related to *Fomitiporia mediterranea*.

## 1. Introduction

*Phellinus pomaceus* var. *prunastri* (Pers.) Pat. 1926, a member of the order Hymenochaetales in the Basidiomycota phylum, is a fascinating basidiomycete fungus that occupies a distinct ecological niche (Markakis et al. 2017). It is predominantly found in forest ecosystems, where it thrives on decaying hardwood trees, particularly oaks (Markakis et al. 2017). The wood-decaying fungus *Phellinus pomaceus* is vital for decomposing woody biomass, thereby influencing soil fertility and the overall health of forest ecosystems. Moreover, interest in *P. pomaceus* in biotechnology has been sparked by its potential utility across diverse industries (Saxena et al. 1998; Sułkowska-Ziaja et al. 2017, 2021). The production of bioactive compounds, including enzymes, secondary metabolites, and polysaccharides, has the potential to serve as a reservoir of antibacterial agents, antioxidants, and immunomodulators in medicine. The bioactive compounds produced by *P. pomaceus* have been widely used in various industries such as biofuels, food processing, and textiles (Harper et al. 1989, 1990, 2001).

The mitochondrial genome in eukaryotes is indispensable for overseeing the growth, development, cellular homeostasis, and the capacity to react to environmental stimuli (Ernster and Schatz 1981; McBride et al. 2006; Murphy 2009). The mitochondrial genome has been proposed as a valuable resource for investigating fungal phylogeny (Xu and Wang 2015; Li et al. 2022a, 2022b, 2023). The mitochondrial genome features of members within the *Phellinus* genus remain poorly understood, with only two mitochondrial genomes documented to date (Lee et al. 2019). This study introduces the initial comprehensive mitochondrial genome of *P. pomaceus*, enhancing the comprehension of the genomic characteristics within this important fungal genus.

## 2. Materials and methods

### 2.1. Sample collection

**Figure 1.**
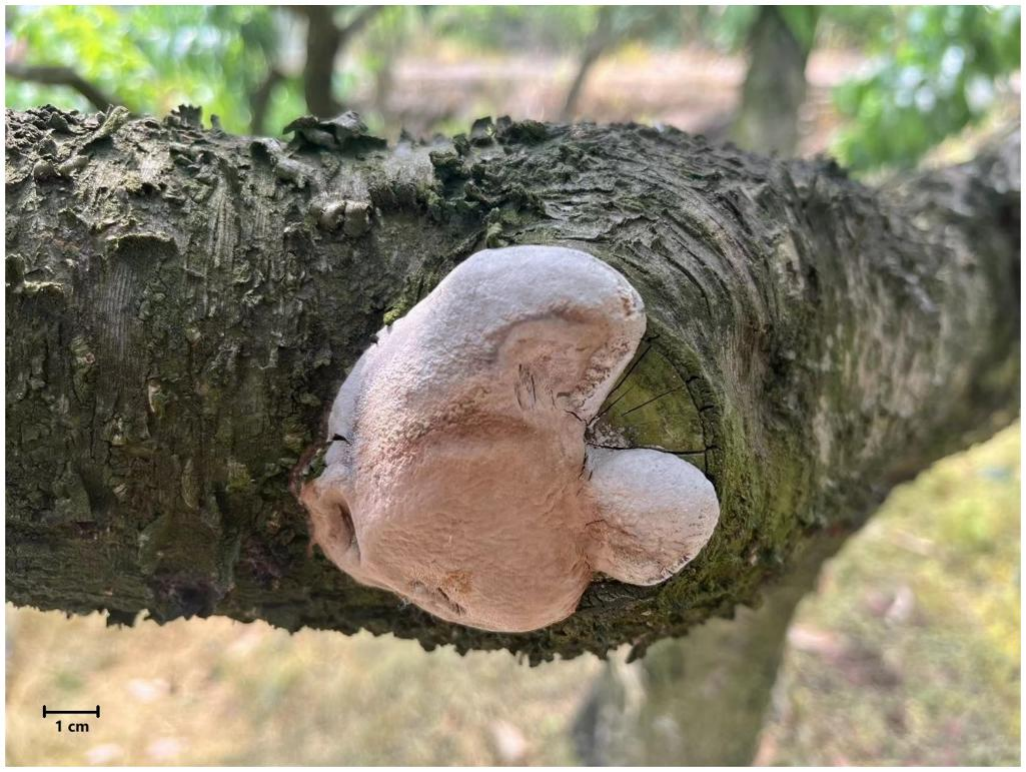
Morphology of *Phellinus pomaceus* fruiting bodies. A photo of the species was taken by Wei Gao using a camera (Canon EOS 5D Mark IV, Canon Inc., Japan).

A *P. pomaceus* sample was collected from a peach tree in Chengdu, Sichuan, China (104.18E, 30.56°N), in 2024. The identification of the samples was based on morphological analysis and nuclear genomic molecular markers, including ITS, translation elongation factor 1-α gene (tef1α), and partial nuclear ribosomal large subunit (nrLSU), as established in earlier studies (Decock et al. 2005; 2006; Han et al. 2016; Zhou et al. 2016; Cho et al. 2023). A specimen was deposited at the Culture Collection Center of Chengdu University under the voucher number Ppo1. For additional information, please contact Wei Gao at gaowei@cdu.edu.cn (Figure 1).

### 2.2. Mitochondrial genome assembly and annotation

We assembled and annotated the mitochondrial genome using the methods described by previous researchers (He et al. 2024). Briefly, the DNA extraction process for *P. pomaceus* fruiting bodies involved using a fungal DNA extraction kit (Norcross, GA, USA). Subsequently, the NEBNext^®^ Ultra^™^ II DNA Library Prep Kit (NEB, Beijing, China) was used for sequencing library preparation following the manufacturer’s instructions. Whole-genome sequencing was carried out using the Illumina HiSeq 2500 platform (Illumina, San Diego, CA, USA). To ensure data accuracy, low-quality sequences were removed using ngsShoRT (Chen et al. 2014) and adapter reads were eliminated using AdapterRemoval v2 (Schubert et al. 2016). The mitochondrial genome of *P. pomaceus* was *de novo* assembled using NOVOPlasty version 4.3.3, with a k-mer size of 32 (Dierckxsens et al. 2017). The annotation of the mitochondrial genome was performed on the basis of established protocols (Li et al. 2019; 2020, 2023), which utilize the MFannot tool (https://megasun.bch.umontreal.ca/apps/mfannot/) (Valach et al. 2014) and MITOS2 (Bernt et al. 2013). Protein-coding genes (PCGs) or open reading frames (ORFs) were annotated or validated *via* BLASTP searches against the NCBI nonredundant protein sequence database (Bleasby and Wootton 1990). The presence of tRNA genes in the mitochondrial genome of *P. pomaceus* was verified *via* the use of tRNAscan-SE v1.3.1 (Lowe and Chan 2016). The PMGmap online web tool was utilized for visualizing gene structures containing introns and graphically representing the mitochondrial genome (http://www.1 kmpg.cn/pmgmap) (Zhang et al. 2024).

### 2.3. Phylogenetic analysis

The phylogenetic tree was constructed according to previously established methods (Li et al. 2020, 2021, 2022a, 2022b). The alignment of individual mitochondrial genes, excluding intron regions, was conducted *via* MAFFT v7.037 software (Katoh et al. 2019). We merged the aligned mitochondrial genes *via* SequenceMatrix v1.7.8, which led to the creation of a unified mitochondrial dataset (Vaidya et al. 2011). To determine potential phylogenetic discrepancies among diverse mitochondrial genes, an initial partition homogeneity test was performed with PAUP v 4.0b10 (Swofford 2002), according to the established literature (Xiang et al. 2013). The combined mitochondrial dataset’s optimal partitioning schemes and evolutionary models were identified *via* PartitionFinder 2.1.1 (Lanfear et al. 2017). The construction of phylogenetic trees *via* the Bayesian inference method was carried out with MrBayes v3.2.6 software (Ronquist et al. 2012). When the BI analysis was conducted, two independent runs with four chains (three heated and one cold) each were conducted simultaneously for 2 × 10^6^ generations. Each run was sampled every 1000 generations. The first 25% of the samples were discarded as burn-in, and the remaining trees were used to calculate Bayesian posterior probabilities (BPP) in a 50% majority rule consensus tree.

## 3. Results

**Figure 2.**
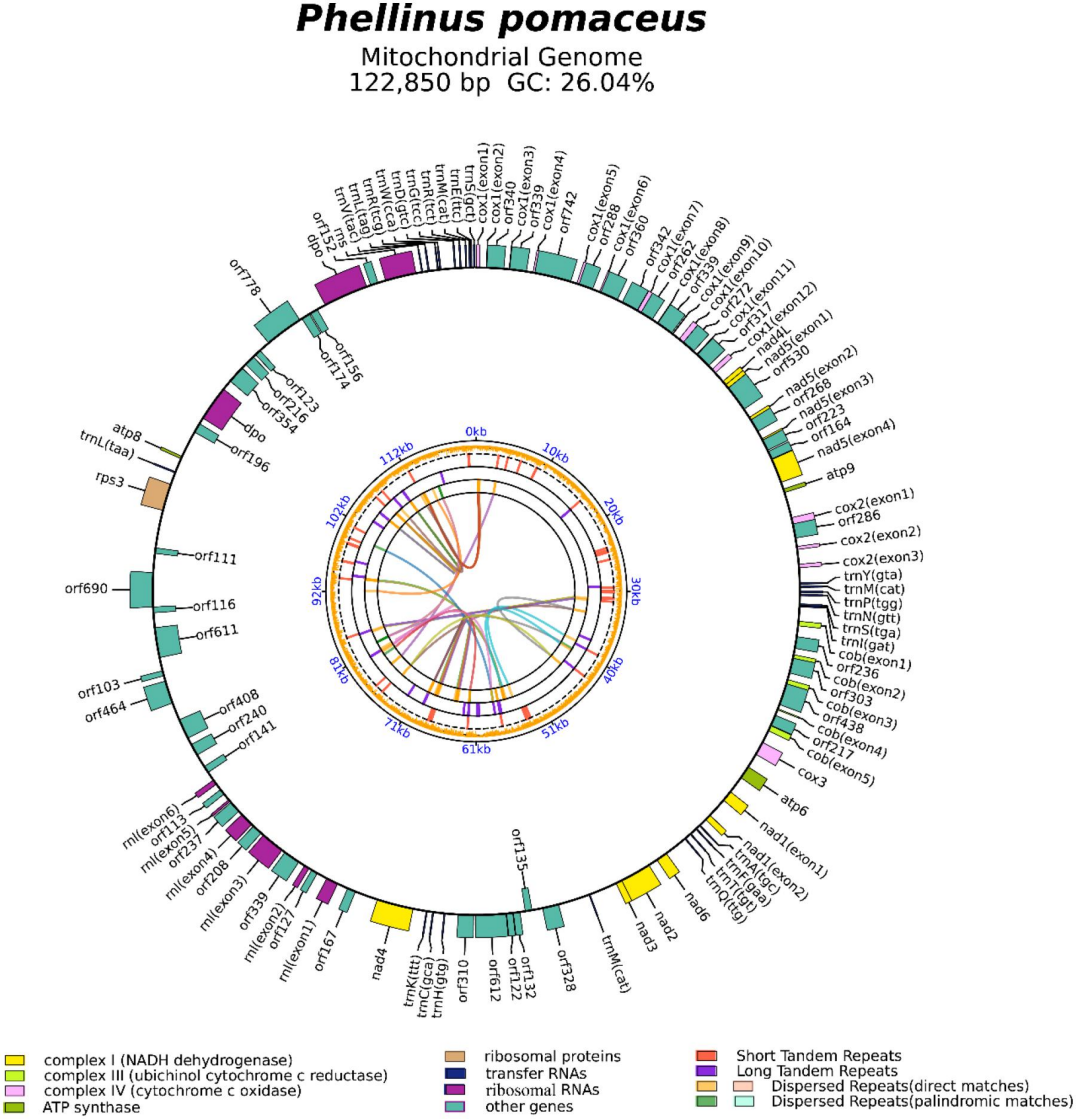
Circular mitochondrial genome map of *Phellinus pomaceus*. Different color blocks represent different genes. Genes located in the outer loop indicate that they are on the direct strand, while genes located inside the loop indicate that they are on the reverse strand. The internal loop represents the repetitive sequence within the mitochondrial genome.

**Figure 3.**
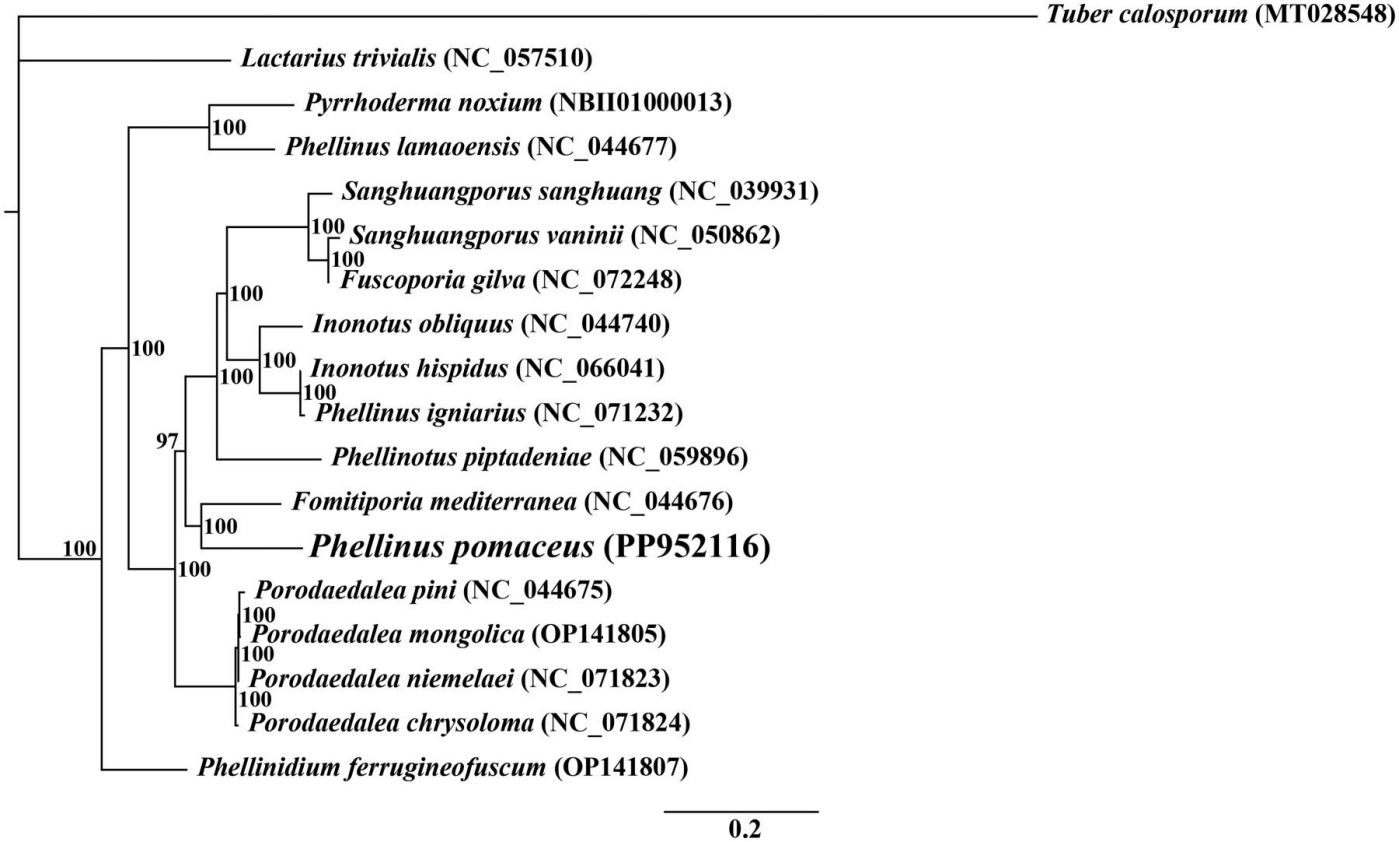
Bayesian inference (BI) tree generated from 14 concatenated mitochondrial protein-coding genes (*atp6*, *atp8*, *atp9, cob, cox1, cox2, cox3, nad1, nad2, nad3, nad4, nad4L, nad5,* and *nad6*) from *Phellinus pomaceus* and 17 other fungal species. *Tuber calosporum* (MT028548) was used as the outgroup (Li et al. 2020). the accession numbers of the sequences were as follows: *Phellinium ferrugineofuscum* (OP141807), *Fomitiporia mediterranea* (NC_044676) (Lee et al. 2019), *fuscoporia gilva* (NC_072248), *sanghuangporus sanghuang* (NC_039931) (Han et al. 2018), *inonotus hispidus* (NC_066041), *Porodaedalea mongolica* (OP141805), *Phellinus* igniarius (NC_071232), *lactarius trivialis* (NC_057510) (Shao et al. 2020), *Phellinus pomaceus* (PP95216), *Porodaedalea pini* (NC_044675) (Lee et al. 2019), *phellinotus piptadeniae* (NC_059896) (Araújo et al. 2021), *pyrrhoderma noxium* (NBII01000013) (Chung et al. 2017), and *Porodaedalea niemelaei* (NC_071823).

The average depth of the coverage-depth map is 1376.88×, as shown in Figure S1. The mitochondrial genome of *P. pomaceus* covers 122,850 bp and has a GC content of 26.04%. The gene structures with introns are depicted in Figure S2. Within *P. pomaceus*, the mitochondrial genome is composed of 36.70% adenine, 13.86% guanine, 37.26% thymine, and 12.18% cytosine. Examination of the mitochondrial genome of *P. pomaceus* revealed 65 open-reading frames, including 15 core protein-coding genes (*cox1*, *cox2*, *cox3*, *atp6*, *atp8*, *atp9*, *cob*, *nad1*, *nad2*, *nad3*, *nad4*, *nad4L*, *nad5*, *nad6*, and *rps3*), along with 26 free-standing ORFs and 24 intronic ORFs, as illustrated in Figure 2. Within the mitochondrial genome of *P. pomaceus*, there are 26 introns, comprising 13 Group IB, 7 Group IA, 1 Group I (derived), 1 Group IC2, 2 Group ID, and 2 unknown types. Intronic ORFs within most introns typically encode LAGLIDADG or GIY-YIG homing endonucleases. The mitochondrial genome of *P. pomaceus* harbors two ribosomal RNA genes (*rns* and *rnl*) and 25 transfer RNA genes. Phylogenetic analysis revealed the closest phylogenetic relationship of *P. pomaceus* to *Fomitiporia mediterranea* (Lee et al. 2019), as depicted in Figure 3.

## 4. Discussion and conclusion

The utilization of the mitochondrial genome can lead to a more comprehensive understanding of the phylogenetic relationships between species (Zhang et al. 2020; Ren et al. 2021; Zhang et al. 2022, 2023). The absence of a mitochondrial reference genome for *P. pomaceus* impedes the use of mitochondrial genomes in classifying and investigating the phylogenetic relationships among Hymenochaetales fungi (Lee et al. 2019). In this study, we sequenced the entire mitochondrial genome of a *Phellinus* species, revealing a length of 122,850 bp with a GC content of 26.04%. The genome consists of 15 core protein-coding genes (PCGs), 26 independent ORFs, 24 intronic ORFs, 25 tRNAs, and 2 rRNA genes. The application of the BI method in phylogenetic analysis revealed that *P. pomaceus* was most closely associated with *Fomitiporia mediterranea* among 17 fungal species from Basidiomycota (Lee et al. 2019), with robust support for major clades. This study contributes to our understanding of the differentiation among *Phellinus* species, as well as the evolutionary patterns and diversity of mitochondria within this important fungal genus (Rajchenberg et al. 2015; Han et al. 2016; Feng et al. 2024).

## Supplementary Material

Supplementary figure.docx

## Data Availability

The genome sequence data that support the findings of this study are openly available in the NCBI GenBank at https://www.ncbi.nlm.nih.gov/under accession no. PP952116. The associated BioProject, SRA, and Bio-Sample numbers are PRJNA1128797, SRR29589343 and SAMN42101229, respectively.
