# Supplementary material for: The first complete mitochondrial genome of Phellinus pomaceus var. prunastri (Pers.) Pat. 1926 (Hymenochaetales: Hymenochaetaceae) and phylogenetic analysis
Source: Mitochondrial DNA B Resour. 2024 Dec 8;9(12):1674–8. doi: 10.1080/23802359.2024.2438275 (PMC11632937; doi:10.1080/23802359.2024.2438275)
Supplement: Supplementary figure.docx [file TMDN_A_2438275_SM0224.docx]

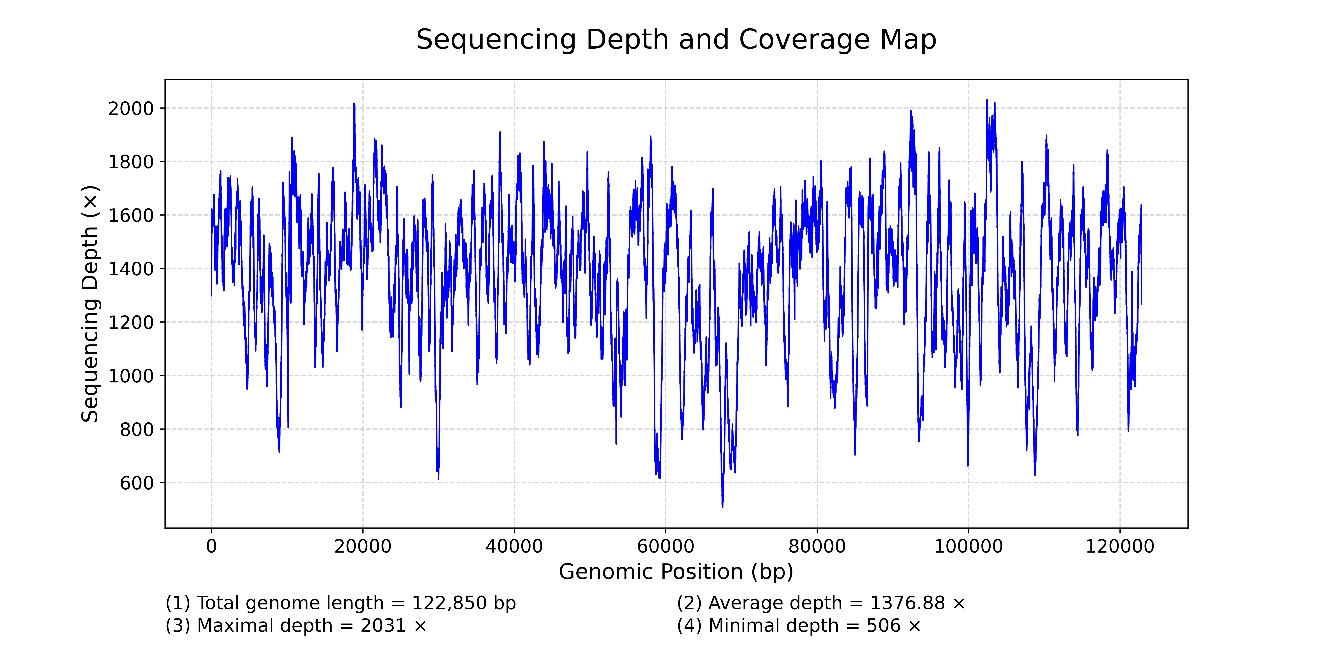


**Figure S1** Sequencing depth and coverage map of *Phellinus pomaceus* mitochondrial genome.


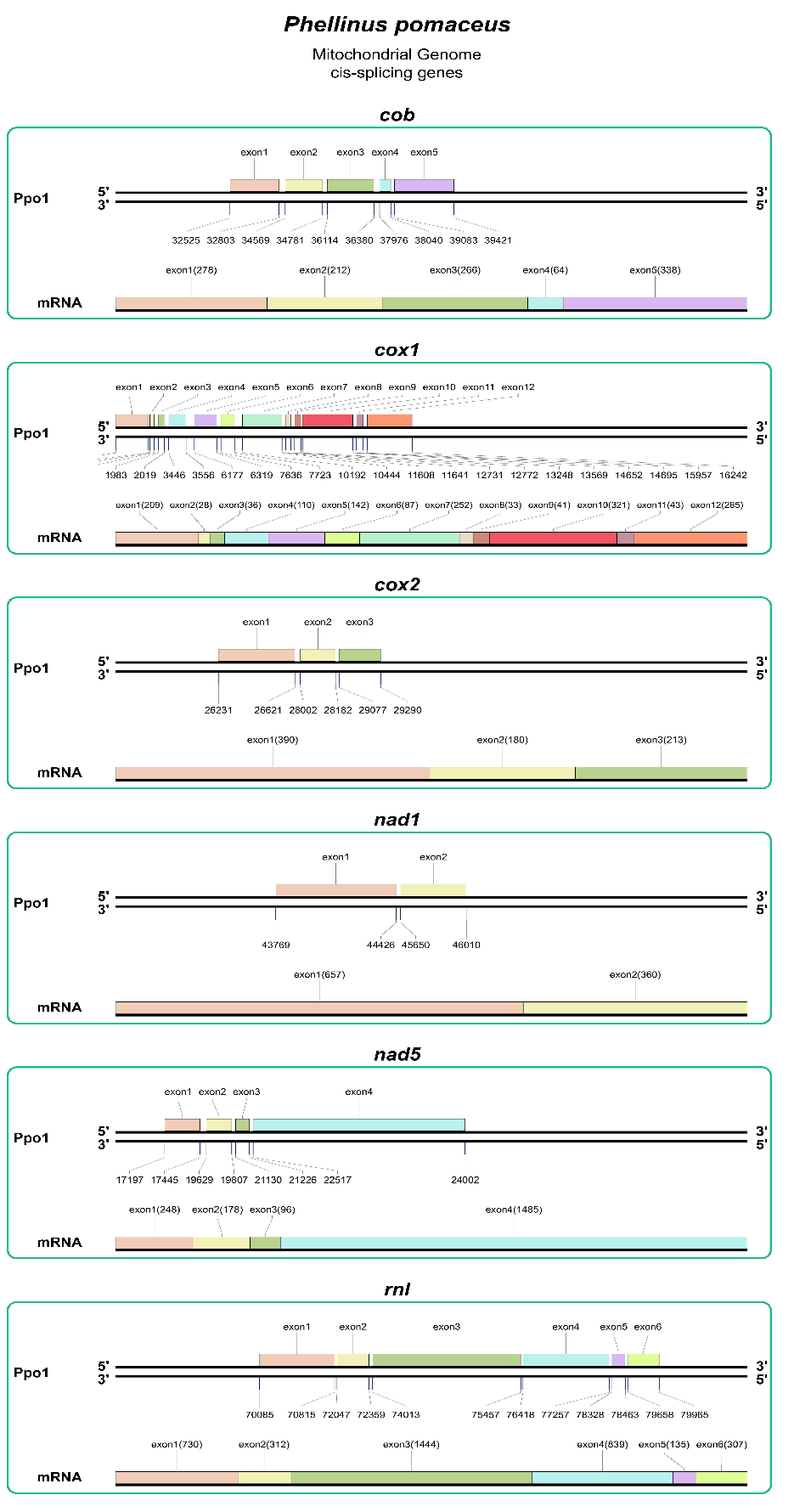


**Supplementary Figure S2** Cis-splicing genes of the *Phellinus pomaceus* mitochondrial genome.
